# Diffusion tensor imaging of the brain white matter microstructure in patients with chronic kidney disease and its correlation with cognition

**DOI:** 10.3389/fneur.2022.1086772

**Published:** 2022-12-15

**Authors:** Chaoyang Zhang, Huan Yu, Yan Cai, Ning Wu, Shuang Liang, Chun Zhang, Zhiyu Duan, Zhou Zhang, Guangyan Cai

**Affiliations:** ^1^Department of Nephrology, General Hospital of the Chinese People's Liberation Army, Beijing, China; ^2^Department of Radiology, Liangxiang Hospital, Beijing, China; ^3^Department of Nephrology, The Affiliated Hospital of Yangzhou University, Yangzhou, Jiangsu, China; ^4^Department of Medical Imaging, Yanjing Medical College, Capital Medical University, Beijing, China

**Keywords:** chronic kidney disease, dialysis, brain white matter, diffusion tensor imaging, cognition decline

## Abstract

**Purpose:**

In individuals with chronic kidney disease (CKD), neurological damage is commonly observed. This neurodegeneration is closely linked to microstructural damage to the brain white matter due to the high incidence of cognitive dysfunction. However, the specific pathogenesis of CKD nephropathy caused by cognitive system developmental disorders remains unclear. This study aimed to examine the correlation between cognitive impairment and diffusion parameters obtained on diffusion tensor imaging (DTI) of abnormal white matter tracts in CKD patients.

**Methods:**

Sixty-four patients with CKD were divided into the non-dialysis-dependent CKD (NDD-CKD) group (*N* = 26) and dialysis-dependent CKD (DD-CKD) group (*N* = 38) according to the estimated glomerular filtration rate, whereas 43 healthy control subjects (normal control [NC]) were included and underwent cranial magnetic resonance imaging during the same period. Differences in the abnormal white matter microstructure and correlations between them and cognitive scores were assessed using several parameters between the groups.

**Results:**

There were more extensive peri-lesions and distant white matter microstructural changes in the DD-CKD and NDD-CKD groups than in the NC group. DTI diffusion parameters in abnormal white matter regions were associated with impaired cognitive function in CKD patients. The DD-CKD group had worse cognitive function and more severe microstructural damage in the cerebral white matter than the NDD-CKD group.

**Conclusion:**

CKD patients showed cognitive impairment and changes in the brain white matter microstructure; CKD can lead to extensive white matter tract damage. Additionally, diffusion parameters can be used as a complement to describe structural brain damage in CKD patients.

## Introduction

With the rapid, healthy, and stable development of China's socialist market economy with distinctive characteristics, people's ideological and spiritual quality and quality of life are increasing, which is accompanied by an increased incidence of CKD. Unfortunately, the resulting complications are not negligible and have invoked widespread concern (1, 2). Among these, potential cognitive and psychological impairment (cognitive impairment, CI) is one of the most important psychological problems that affect the quality of life and wellbeing of patients with CKD (3). Patients with CKD are at a higher risk of cognitive impairment, and older patients on dialysis have the highest absolute risk (4). Neuroimaging studies have shown that patients with CKD tend to have structural and functional changes, such as white matter hyperintensities, asymptomatic stroke, and brain atrophy (5, 6). The white matter of the brain acts as a relay station for the central nervous system and is responsible for the exchange of information and communication between different gray matter areas (7, 8). At present, there are only few studies on the microstructure of the cerebral white matter in CKD patients (9, 10). Beam-based spatial statistics (TBSS) is a more accurate and comprehensive method for assessing the microstructural changes in whole-brain white matter. Because cognitive impairment in different regions can be reflected by neuroimaging abnormalities, it is important to understand the interaction between cognitive impairment and structural brain tissue damage caused by different degrees of renal damage to minimize the risk of neurological injury in CKD patients (11). In this study, we aimed to investigate the correlation between diffusion parameters obtained from abnormal white matter bundle DTI and cognitive function in CKD patients. To achieve this goal, TBSS based on DTI data was used to construct a skeleton of the white matter of the brain in patients with CKD. Diffusion parameters were analyzed to determine the white matter microstructure. These parameters of the combined DTI can then be used to accurately assess cyclical changes in the white matter microstructure in the human brain and their direct correlation with cognitive system function.

## Materials and methods

### Clinical data of the subjects

This study was conducted following the recommendations of patients with CKD were selected from Liangxiang Hospital of Capital Medical University during January 2021 to December 2021, and informed consent was obtained from all subjects. Age, sex, and general demographic data of the subjects were collected through a review of medical records and interviews. Estimated glomerular filtration rate (eGFR) was calculated using the modified Cockcroft-Gault formula: eGFR = 186 × (SCr/88.4)-1.154 × (age)-0.203 × (0.742, female). Patients were grouped according to the eGFR values: eGFR of < 15 mL/(min 1.73 m^2^) in the DD-CKD group; eGFR of 15–60 mL/(min 1.73 m^2^), in the DD-CKD group; and eGFR of ≥60 mL (min 1.73 m^2^) in the NDD-CKD group.

The inclusion criteria were as follows: (1) CKD patients who met the National Kidney Foundation–Kidney Disease Outcomes Quality Initiative diagnostic criteria; (2) age ≥18 years; (3) receiving maintenance hemodialysis or continuous ambulatory peritoneal dialysis for >3 months; and (4) no infection or other complications within the last 3 months. The exclusion criteria were (1) contraindication to magnetic resonance imaging (MRI) examination; (2) previous neurological and psychiatric diseases; and (3) history of the tumor. During the same period, 43 age- and sex-matched normal control (NC) subjects were included. This study was approved by the Ethics Committee of Liangxiang Hospital, and all participants included in the study had signed informed consent at the time of registration.

### Cognitive function tests

Psychological function test is based on the original modern method of the neuropsychological function test, a type of modern psychological function test that is suitable for the assessment of the human brain. All participants underwent cognitive function tests immediately after enrollment, including the Chinese version of the Simple Mental State Examination (MMSE) and the Beijing version of the Montreal Cognitive Assessment (MoCA), to assess subjects' cognitive function in which an MMSE score of ≤ 18 was classified as dementia, 19–26 as moderate-to-mild cognitive impairment, and ≥27 as normal. The MoCA scale includes attention, concentration, executive function, memory, language, visual structure skills, abstract thinking, calculation, and orientation skills, with a total score of 30. Data collection and collation were performed by the investigators. The questionnaire requirements were explained in the same language, and consistent explanations were used for resolving any doubts to avoid possible understanding bias when filling out the questionnaires. The valid return rate of the questionnaires was 100%, and data collation was performed on the day the questionnaires were collected and checked by two people. It includes the Traditional Chinese version of the MMSE and Beijing Chinese version of the MoCA.

### MRI examination

We used the MRI scanner MAGENTOM Skyra 3.0 T using a 32-channel two-phased array magnetic head coil. All participants underwent the following examinations: (1) three-dimensional T1-weighted structural imaging with the following scan parameters: MP-RAGE sequence, repetition time (TR) = 2,000 ms, inversion time (TI) = 880 ms, echo time (TE) = 2.01 ms, flip angle (FA) = 8°, matrix = 256 × 256, field of view (FOV) = 256 × 256 mm^2^, sagittal total thickness = 208 mm, and thickness = 1 mm; (2) a DTI scan sequence with the following sweep parameters: a single-excitation spin echo plane imaging sequence with DTI data obtained in 30 independent non-collinear directions at b = 1,000 and 0 s/mm^2^, respectively, TR = 9,200 ms, TE = 70 ms, FA = 90°, matrix = 112 × 112, FOV = 224 × 224 mm^2^, layer thickness = 2 mm, number of layers = 72, fat suppression on, and parallel imaging factor = 2; and (3) a field map scan for the correction of echo planar imaging deformation with the following scan parameters: double-echo gradient echo sequence with TR = 400 ms, TE = 4.92/7.38 ms, FA = 60°, matrix = 64 × 64, FOV = 224 × 224 mm^2^, layer thickness = 4 mm, and number of layers = 36.

During functional MRI data acquisition, all subjects were asked to close their eyes, lie down, relax, and remain as awake and calm as possible at rest.

### Image analysis

#### Data preprocessing

The diffusion-weighted imaging data of each participant were preprocessed using the following steps: image denoising, removal of Gibbs ring artifacts, motion and eddy current distortion correction, and bias field correction.

#### Diffusion model fitting

Voxel-level calculations of diffusion metrics were performed using FSL to derive fractional anisotropy (FA), mean diffusivity (MD), axial diffusivity (AD), and radial diffusivity (RD) images.

#### TBSS

All FA images were converted to an MNI standard image space using a non-linearized registration conversion method, and then, we recreated the average accuracy FA image maps for each subject, which represent the centers of all nerve bundles common to all three groups of subjects. Finally, all individual parameter values (including FA, MD, AD, and RD) were projected onto the population skeleton for further statistical analysis.

### Statistical analyses

Demographic, clinical, and neuropsychological data were analyzed using SPSS 25.0. Differences in demographic and clinical neuropsychological data were analyzed. Comparative images of demographic and clinical data among the three groups were analyzed using *t*-tests and χ^2^ tests.

A two-sample *t*-test was performed using DPABI software to compare the NC and NDD-CKD groups, NC and DD-CKD groups, and NDD-CKD and DD-CKD groups sequentially for understanding differences in skeletonized FA, FA skeleton as a mask; after regressing off the effects of age and multiple effects of sex, Gaussian random field correction calculations (Gaussian random field, grf) were made for the correction of multiple comparison values, and voxel generation levels significantly increased *p* < 0.001 for the correction of generating voxel clusters. Brain regions with a cluster-level significance of *p* < 0.05 were defined as regions of interest, and their FA values were extracted and used for correlation analysis with cognitive scales; the same approach was used for statistical analyses of MD, AD, and RD.

## Results

### Demographic and cognitive outcomes

In terms of the demographic and clinical characteristics (e.g., age and sex) of the NDD-CKD and NC groups, there were no significant differences. Patients in the NDD-CKD group had a higher prevalence of hypertension, diabetes, and dyslipidemia; lower hemoglobin, protein, albumin, and phosphate levels and red blood cell counts; and higher blood urea nitrogen and creatinine levels than those in the NC group. Compared with patients with NDD-CKD, those with DD-CKD had worse cognitive function. The characteristics of patients in each group are shown in Tables 1–3.

**Table 1:** Demographic of the CKD and NC groups.

	**CKD (*****N** **=*** **64)**		**NC (*N =* 43)**	***T*****/*****X***^**2**^ **value**			***P*** **-value**		
	**NDD-CKD(*N =* 26)**	**DD-CKD(*N =* 38)**		**A**	**B**	**C**	**A**	**B**	**C**
Age (years)	56.88 ± 11.70	58.06 ± 11.07	55.64 ± 9.70	0.47	−0.99	−0.39	0.64	0.32	0.69
Male, *n* (%)	17 (65.38)	18 (58.06)	28 (66.67)	0.01	0.57	0.32	0.91	0.45	0.57
Duration (years)	3.00 ± 2.99	5.00 ± 4.50	NA	NA	NA	−1.93	NA	NA	0.05
Hypertension, *n* (%)	23 (88.46)	28 (90.32)	5 (11.90)	38.86	44.28	0.05	0.00^**^	0.00^**^	0.82
Diabetes, *n* (%)	19 (73.08)	18 (58.06)	1 (2.38)	38.65	28.72	1.40	0.00^**^	0.00^**^	0.24
Dyslipidemia, n (%)	17 (65.38)	23 (74.19)	2 (4.76)	29.31	38.18	0.52	0.00^**^	0.00^**^	0.47

**P < 0.01.

A: Comparison between the NC and NDD-CKD groups (NC vs. NDD-CKD).

B: Comparison between the NC and DD-CKD groups (NC vs. DD-CKD).

C: Comparison between the NDD-CKD and DD-CKD groups (NDD-CKD vs. DD-CKD).

**Table 2:** Clinical characteristics of the CKD and NC groups.

	**CKD (*****N =*** **64)**		**NC (*N =* 43)**	***T*****/*****X***^**2**^ **value**			***P*** **-value**		
	**NDD-CKD(*N =* 26)**	**DD-CKD(*N =* 38)**		**A**	**B**	**C**	**A**	**B**	**C**
Hemoglobin, g/dL	104.85 ± 23.66	112.61 ± 18.24	134.95 ± 16.27	−5.71	5.51	−1.40	0.00**	0.00**	0.17
Erythrocyte pressure, %	31.85 ± 6.61	35.06 ± 5.78	40.45 ± 4.24	−5.92	4.60	−1.95	0.00**	0.00**	0.06
Protein, g/dL	59.96 ± 9.55	67.42 ± 7.33	65.26 ± 5.50	−2.54	−1.44	−3.31	0.02*	0.16	0.002**
Albumin, g/dL	32.50 ± 6.63	35.19 ± 4.62	38.24 ± 2.72	−4.20	3.27	−1.75	0.00**	0.002**	0.09
Blood urea nitrogen, mg/dL	14.30 ± 11.47	18.96 ± 5.02	5.02 ± 1.36	4.11	−15.046	−1.920	0.000**	0.000**	0.064
Creatinine, mg/dL	392.62 ± 328.65	969.35 ± 297.85	67.26 ± 16.41	5.04	−16.844	−6.946	0.000**	0.000**	0.000**
Calcium, mg/dL	2.19 ± 0.20	2.37 ± 0.19	2.30 ± 0.11	−2.54	−1.71	−3.36	0.02*	0.09	0.001**
Phosphate, mg/dL	1.45 ± 0.48	1.79 ± 0.73	1.09 ± 0.27	3.97	−5.06	−2.01	0.00**	0.00**	0.049*

*P < 0.05 and **P < 0.01.

**Table 3:** Cognitive scores of the CKD and NC groups.

	**CKD (*****N** **=*** **64)**		**NC (*N =* 43)**	***T*****/*****X***^**2**^ **value**			***P*** **-value**		
	**NDD-CKD(*N =* 26)**	**DD-CKD(*N =* 38)**		**A**	**B**	**C**	**A**	**B**	**C**
MMSE score	27.00 ± 1.26	22.39 ± 2.75	28.38 ± 1.27	−4.37	11.27	8.34	0.00**	0.00**	0.00**
MoCA score	25.46 ± 2.14	23.55 ± 2.28	27.29 ± 1.60	−3.75	7.83	3.25	0.001**	0.00**	0.002**

**P < 0.01.

### Differences in diffusion parameters derived from diffusion tensor imaging

#### Comparative analysis of damaged white matter areas between the NDD-CKD and NC groups

The results of two-sample *t*-tests showed that the NDD-CKD group exhibited white matter damage in several brain regions, as demonstrated by decreased FA and increased MD, AD, and RD, as well as significant intergroup differences in white matter tract DTI diffusion parameters.

**Figure 1:**
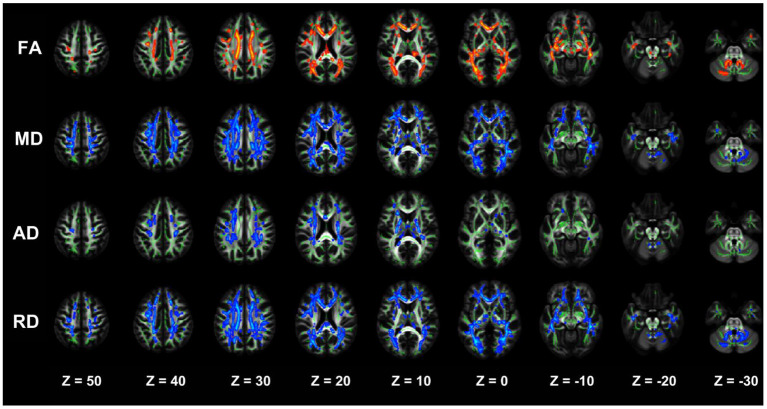
TBSS analysis showing abnormal white matter bundles in areas with differences in DTI parameters, including the FA, MD, AD, and RD, between the NDD-CKD and NC groups. Red color indicates significantly increased white matter bundle parameters, and blue color indicates significantly decreased white matter bundle parameters. Green color represents the mean FA frame for all subjects. FA, fractional anisotropy; MD, mean diffusivity; AD, axial diffusivity; RD, radial diffusivity; TBSS, tract-based spatial statistics; DTI, diffusion tensor imaging.

The NDD-CKD group showed significant changes in the microstructure of the white matter in the areas above and below the wide bilateral screen compared with the NC group (Figure 1, Table 4). DTI_TBSS analysis showed that the right thalamus retrorelene (PTR_R), right sagittal layer (SS_R), corpus callosum (BCC), left anterior radiation crown (ACR_L), left upper radiation crown (SCR_L), and callosum pressure (SCC) were characterized by decreased FA and elevated MD and RD. The posterior left thalamic radiation (PTR_L) showed a decrease in FA and an increase in RD. The posterior part of the right inner capsule lens (RLIC_R), posterior part of the right radiative crown (PCR_R), superior longitudinal tract (SLF_R, SLF_L), and mid-cerebellar foot (MCP) showed elevated MD and RD. The right hind limb of the inner capsule (PLIC_R) is a manifestation of MD elevation. The genu of the corpus callosum (GCC), right external capsule (EC_R), left posterior corona radiata (PCR_L), left inferior cerebellar peduncle (ICP_L), and right anterior corona radiata (ACR_R) showed increased RD.

**Table 4:** Regions with significant differences in abnormal white matter bundles caused by different DTI parameters in the NDD-CKD and NC groups (only clusters larger than 500 voxels are shown).

	**Cluster**	**Number of voxels**	**Peak T value**	**Peak MNI coordinates**			**Structure name**
				**x**	**y**	**z**	
FA	1	1,402	6.631	28	−75	3	PTR_R, SS_R
	2	934	6.510	−34	−63	4	PTR_L
	3	841	5.633	2	−24	23	BCC, ACR_L, SCR_L, SCC
MD	1	4,967	−7.206	39	−67	−6	SS_R, RLIC_R, SCC, BCC, PTR_R, PLIC_R, PCR_R, SLF_R
	2	1,718	−6.917	19	−44	−24	MCP
	3	1,303	−6.358	−18	−1	40	SCR_L, ACR_L, BCC
	4	930	−5.967	−35	1	27	SLF_L
RD	1	6,062	−7.664	33	−63	2	BCC, PTR_R, ACR_L, SS_R, SCC, SCR_L, RLIC_R, PCR_R, GCC, SLF_R, EC_R, PCR_L
	2	1,555	−6.561	−28	−65	21	PTR_L
	3	1,098	−6.344	16	−49	−29	MCP
	4	748	−7.048	−8	−52	−19	MCP, ICP_L
	5	595	−5.908	−35	1	27	SLF_L
	6	541	−5.531	14	31	−10	ACR_R

#### Comparative analysis of damaged white matter areas between the DD-CKD and NC groups

**Figure 2:**
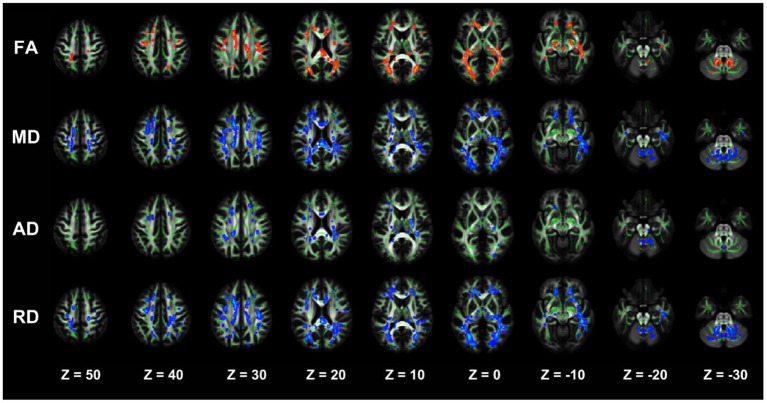
TBSS analysis showing abnormal white matter bundles for DTI parameters, with the FA, MD, AD, and RD difference areas between the DD-CKD and NC groups. Red color indicates significantly increased white matter bundle parameters, and blue color indicates significantly decreased white matter bundle parameters. Green color represents the mean FA frame for all subjects. FA, fractional anisotropy; MD, mean diffusivity; AD, axial diffusivity; RD, radial diffusivity; TBSS, tract-based spatial statistics; DTI, diffusion tensor imaging.

The two-sample *t*-test results of comparisons between the DD-CKD and NC groups showed that the DD-CKD group had white matter damage in several brain regions and reduced FA, as well as elevated MD, AD, and RD. The significantly different white matter tracts between the DD-CKD and NC groups are shown in Figure 2, Table 5. The body of the corpus callosum (BCC), genu of the corpus callosum (GCC), right anterior corona radiata (ACR_R), sagittal stratum (SS_L, SS_R), left retrolenticular part of the internal capsule (RLIC_L), and right posterior thalamic radiation (PTR_R) manifested as decreased FA and elevated MD and RD. The left anterior corona radiata (ACR_L), superior corona radiata (SCR_L, SCR_R), splenium of the corpus callosum (SCC), posterior corona radiata (PCR_L, PCR_R), superior longitudinal fasciculus (SLF_L, SLF_R), and right retrolenticular part of the internal capsule (RLIC_R) manifested as decreased FA and elevated AD, MD, and RD. The left posterior thalamic radiation (PTR_L), fornix/striatum terminalis (Fx/ST_R, Fx/ST_L), and external capsule (EC_R, EC_L) showed a decrease in FA and an increase in RD. The right cerebral peduncle (CP_R) exhibited reduced FA. The posterior limb of the internal capsule (PLIC_R, PLIC_L) manifested as elevated AD and MD. The left anterior limb of the internal capsule (ALIC_L) exhibited elevated AD, MD, and RD. Lastly, the middle cerebellar peduncle (MCP) and superior cerebellar peduncle (SCP_R) showed elevated RD.

**Table 5:** Regions with significant differences between the DD-CKD and NC groups (only clusters larger than 500 voxels are listed).

	**Cluster**	**Number of voxels**	**Peak T value**	**Peak MNI coordinates**			**Structure name**
				**x**	**y**	**z**	
FA	1	8,765	8.36131	−4	−11	26	BCC, GCC, ACR_L, PTR_L, ACR_R, SCR_L, SCC, SCR_R, PCR_L, SS_L, PCR_R, SLF_L, RLIC_L
	2	3,171	7.57058	42	−25	−9	PTR__R, SS_R, Fx/ST_R, PCR_R, RLIC_R, SLF_R, EC_R, CP_R
	3	769	7.76303	−32	−24	−6	Fx/ST_L, SS_L, EC_L, RLIC_L
MD	1	30,667	−7.83861	−8	−14	27	BCC, ACR_L, SCR_L, SLF_L, SLF_R, SCR_R, PCR_R, SCC, PTR_R, PCR_L, ACR_R, SS_R, GCC, RLIC_R, PCR_L, PLIC_R, SS_L, RLIC_L, ALIC_L, PLIC_L
AD	1	1,866	−7.24445	−22	19	32	SCR_L, PLIC_L, ACR_L, ALIC_L
	2	1,759	−6.87289	29	−25	43	PCR_R, PLIC_R, RLIC_R, SCR_R, SLF_R
	3	723	−5.85007	−34	−24	39	PCR_L, SLF_L, SCC
RD	1	30,938	−8.90067	39	−35	−4	BCC, ACR_L, GCC, SCR_L, ACR_R, PTR_R, PTR_L, SLF_L, SCC, PCR_R, PCR_L, SS_R, SLF_R, SCR_R, SS_L, RLIC_R, RLIC_L, Fx/ST_L, EC_L, EC_R, Fx/ST_R, ALIC_L
	2	994	−6.28618	28	−60	−23	MCP, SCP_R
	3	776	−6.39299	29	1	44	SLF_R
	4	666	−5.91248	−19	−60	−30	MCP

PTR, Posterior thalamic radiation (include optic radiation); SS, Sagittal stratum (including the inferior longitudinal fasciculus and inferior fronto-occipital fasciculus); BCC, Body of the corpus callosum; ACR, Anterior corona radiata; SCR, Superior corona radiata; SCC, Splenium of the corpus callosum; RLIC, Retrolenticular part of the internal capsule; PLIC, Posterior limb of the internal capsule; PCR, Posterior corona radiata; SLF, Superior longitudinal fasciculus; MCP, Middle cerebellar peduncle; EC, External capsule; ICP, Inferior cerebellar peduncle; FX/ST, Fornix/striatum terminalis; CP, Cerebral peduncle; ALIC, Anterior limb of internal capsule; SCP, Superior cerebellar peduncle; GCC, Genu of the corpus callosum.

#### Comparative analysis of damaged white matter areas in the NDD-CKD and DD-CKD groups

**Figure 3:**
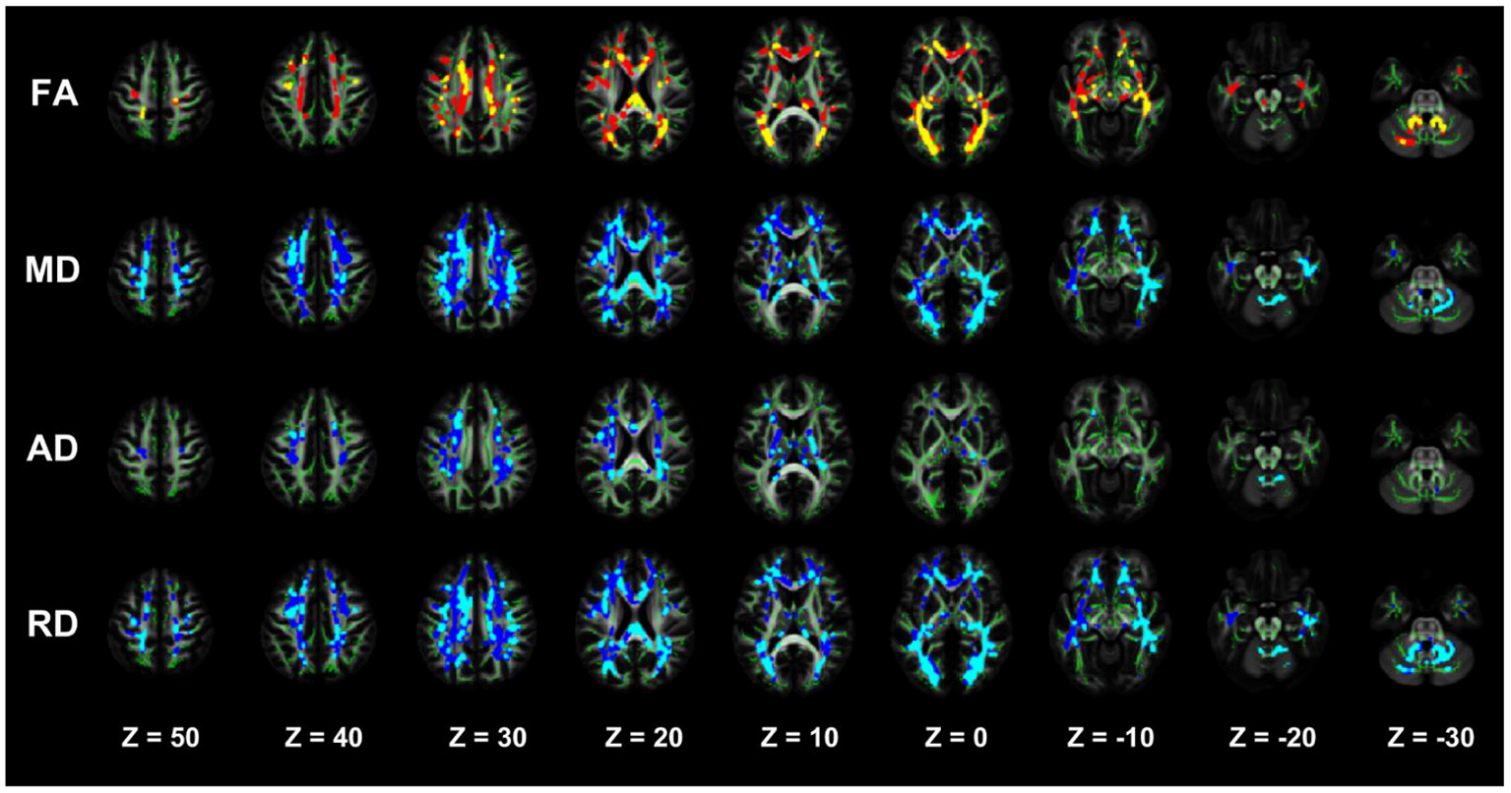
TBSS analysis showing abnormal white matter bundles for DTI parameters, with the FA, MD, AD, and RD difference areas between the NDD-CKD and DD-CKD groups. Yellow or light blue color represents the commonly damaged area, corresponding to *in the table below; red or dark blue color represents the area damaged in the DD-CKD group and not damaged in the NDD-CKD group, corresponding to # in the table below. FA, fractional anisotropy; MD, mean diffusivity; AD, axial diffusivity; RD, radial diffusivity; TBSS, region-based spatial statistics; DTI, diffusion tensor imaging.

The TBSS analysis did not differ significantly in terms of the FA, MD, AD, and RD between the NDD-CKD and DD-CKD groups, although a more lenient correction method was utilized (Table 6, Figure 3).

**Table 6:** Differences in the damaged white matter volume (cm^3^) between the NDD-CKD and DD-CKD groups.

**Volume of the damaged cerebral white matter**	**FA (cm^3^)**	**MD (cm^3^)**	**AD (cm^3^)**	**RD (cm^3^)**
NDD-CKD	64.988	131.284	27.448	130.414
DD-CKD	116.031	211.496	60.319	210.269
NDD-CKD in common with DD-CKD	47.583	105.251	20.652	106.318
DD-CKD is damaged, but NDD-CKD is not	68.448	106.245	39.667	103.951
Damage in the NDD-CKD group but not in the DD-CKD group	17.405	26.033	6.796	24.096

### Correlation analysis of diffusion parameters and cognitive function in damaged brain white matter areas

**Figure 4:**
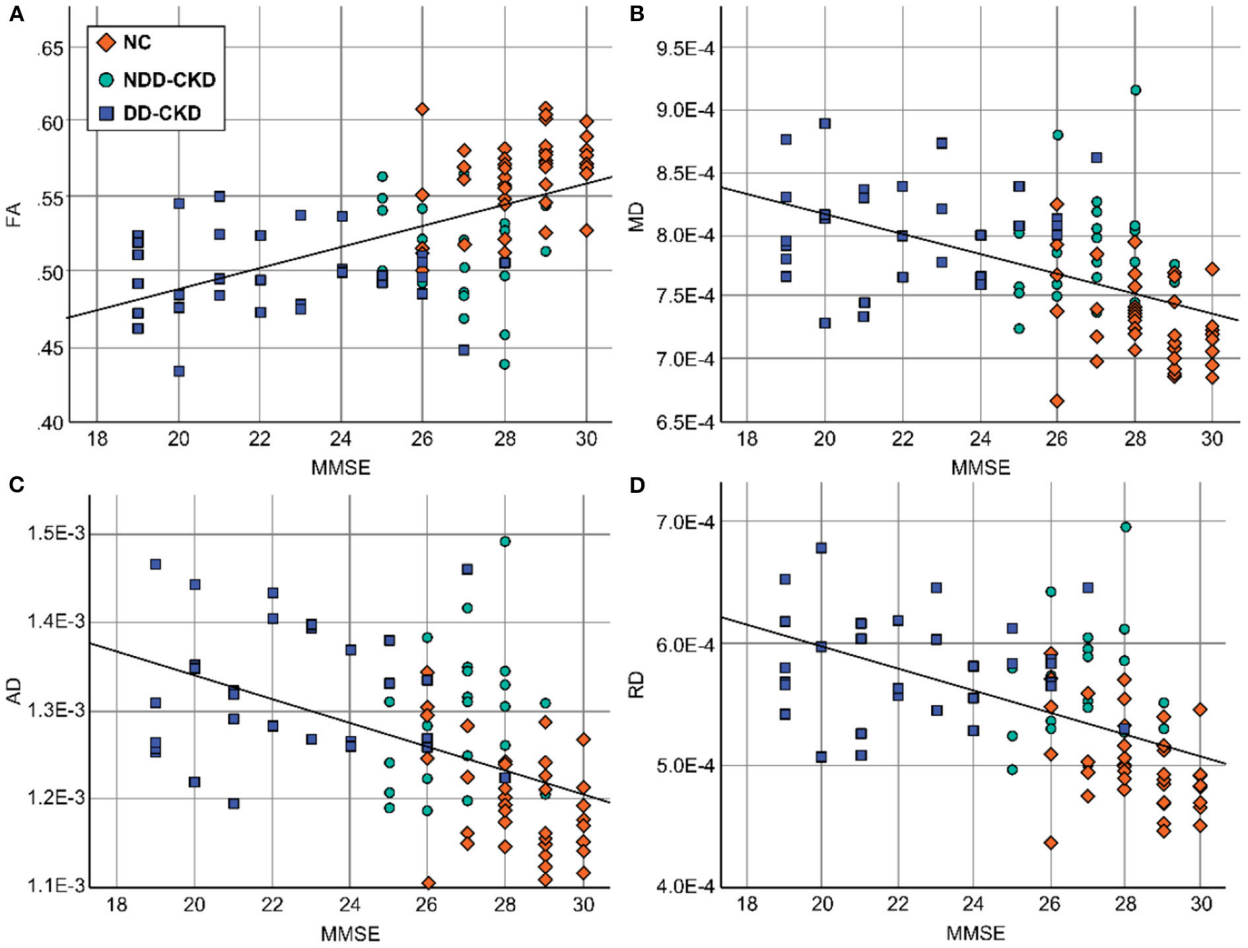
Relationship between diffusion parameters and cognitive function in damaged brain white matter areas. **(A)** The FA values were positively correlated with the MMSE scores. **(B)** The MD values were negatively correlated with the MMSE scores. **(C)** The AD values were negatively correlated with the MMSE scores. **(D)** The RD values were negatively correlated with the MMSE scores. FA, fractional anisotropy; MD, mean diffusivity; AD, axial diffusivity; RD, radial diffusivity; MMSE, Simple Mental State Examination.

Brain white matter microstructural abnormalities were associated with cognitive function in patients with CKD. FA, MD, RD, and RD values were correlated with the cognitive function scale scores. Decreased FA values were positively correlated with the MMSE and MoCA scores, and the MD, RD, and RD values were negatively correlated with the MMSE and MoCA scores (Table 7, Figure 4).

**Table 7:** Correlation analysis of diffusion parameters in damaged brain white matter areas with the MMSE and MoCA cognitive function scores.

		**FA**	**MD**	**AD**	**RD**
MMSE	R	0.546**	−0.0499**	−0.0487**	−0.0525**
	p	3.2E-09	8.2E-08	1.8E-07	1.4E-08
MoCA	R	0.418**	−0.392**	−0.394**	−0.402**
	p	9.3E-06	3.3E-05	3.0E-05	2.1E-05

A p-value of < 0.05 was considered statistically significant. **P < 0.01.

## Discussion

CKD, a group of diseases characterized by eGFR of < 60 mL/(min 1.73 m^2^) or renal injury >3 months owing to different causes, in reference to a much higher risk of cognitive impairment than that in the general population (12). In recent years, studies have reported that the incidence of CKD is increasing and has become an important global public health issue (13). The consequent cardiovascular and cerebrovascular complications have attracted the attention of researchers (14). Almost all CKD patients have a combination of mild-to-moderate cognitive impairment. We found that the MMSE and MoCA cognitive scores of the NDD-CKD, DD-CKD, and NC groups were significantly different according to the questionnaire analysis. Cognitive impairment exhibited cumulative effects at all stages of CKD, an association between renal dysfunction and cognitive impairment as a consequence of CKD, and changes in the microstructure of the brain white matter as a result of CKD. This supports the hypothesis that cerebral white matter microstructural abnormalities affect cognitive function during the progression of CKD. Cerebral white matter lesions underlie its structure, although studies of secondary cerebral white matter microstructural changes at the whole-brain level in CKD patients are uncommon.

DTI imaging is based on traditional diffusion-weighted computer imaging analysis technology, which is an advancement in imaging analysis technology; through imaging analysis of water molecules in different angles and observation of the fine fiber structure, changes in the brain white matter ultrastructure can be observed before tissue morphological changes occur. Because of the large correlation between the severity of cognitive corpus callosum and brain atrophy and the sustained impairment of patients' cognitive system function, the numerical detection of F and FA of the cognitive corpus callosum can be used as a sensitive detection index for the diagnosis of fiber injury damage in the corpus callosum and cerebral white matter to assess whether cognitive system function is impaired in patients. DTI can aid in evaluating abnormal pathological and physiological changes in the tissue microstructure and can detect abnormal changes in the white matter microstructure. Early use of cognitive DTI can detect brain white matter fiber damage at different nerve sites, especially the right upper frontal lobe, left upper and lower temporal lobe, and the left knee of the corpus callosum, which has certain reference significance for early patients to distinguish between individuals with cognitive mild cognitive impairment and those with normal cognitive system function development in later stages.

The corpus callosum comprises white matter tracts that connect the cerebral hemispheres; it plays a crucial role in cognitive functions, such as memory formation and extraction processes (15). When white matter lesions involve the corpus callosum, they may cause extensive disconnections in the cortical and subcortical networks, thus aggravating cognitive decompensation leading to dementia (16). Studies have reported that there is a correlation between the degree of corpus callosum atrophy and cognitive impairment (17–19). For this reason, corpus callosum FA values can be used as a sensitive indicator of white matter fiber damage in the corpus callosum to assess cognitive impairment. Based on the TBSS method, our results confirmed that in the corpus callosum of the NDD-CKD and DD-CKD groups, FA decreased, MD increased, and RD increased in the right anterior corona radiata, sagittal lamina, left internal capsule nucleus pulposus, and right posterior thalamus radiation compared with that of the NC group. The DD-CKD group showed the most extensive lesions. Consistent with the results of the MMSE and MoCA scale analyses, we know that changes in the brain white matter microstructure in the corpus callosum will reduce the remote interactive control of motor, perception, and cognition. The corpus callosum showed reduced FA, elevated MD and RD, and reduced cognitive function scores, suggesting that corpus callosum damage in CKD patients may be related to impaired information processing speed, which also implies that the corpus callosum may play a role in cognitive dysfunction in CKD. Further analysis of the correlations indicated that decreased FA values were positively correlated with the MMSE and MoCA scores, whereas MD, AD, and RD values negatively corresponded to MMSE and MoCA scores. This suggests that white matter lesions, especially corpus callosum lesions, are involved in the development of cognitive impairment in CKD patients.

We also observed that, in addition to the corpus callosum, some specific white matter tracts such as the superior longitudinal tract, inferior longitudinal tract, and inferior frontal-occipital tract also showed a trend of decreased FA and increased MD, AD, and RD in the NDD-CKD and DD-CKD groups, and the extent of lesions was also more pronounced in the DD-CKD group. Damage to these three structures leads to reduced information processing speed and memory capacity. Moreover, damage to the inferior frontal-occipital tract, an important pathway linking the frontal, temporal, and occipital lobes, affects neuromotor function and audiovisual processing. Thalamic radiation may work in the neuropathological mechanisms of executive function and memory, whose damage in microstructures were also consistent with the trend of cognitive impairment in our observation. This suggests an association between damage to the cerebral white matter microstructures and the prevalence of cognitive impairment in CKD. Different concentrations of ions present in the dialysate will affect the osmotic pressure of cerebrospinal fluid and consequently affect the structure and function of the brain tissue. This, in turn, affects cognitive function correlation.

### Limitations

One of the limitations of the study was its single-center cross-sectional observational design, which limited our ability to observe the dynamic course of disease development. In future studies, we will include more subjects and observe them over a longer time period to obtain more accurate and reliable results.

## Conclusion

Many CKD patients experience cognitive dysfunction. In this study, we performed DTI-TBSS analysis to examine the microstructure of the white matter in patients with CKD. We found that DTI parameters provided more comprehensive information and that abnormal white matter bundle parameters were significantly correlated with cognitive function scores. There is some evidence to suggest that large-scale deterioration of the white matter microstructure may contribute to cognitive impairment in CKD. Neuroimaging can help us to determine the progression of the disease course and diffusion parameters, as complementary parameters to structural damage of the brain tissue in CKD patients. This suggests that microstructural changes in the white matter of the brain may cause intra- and inter hemispheric communication disruptions in CKD patients.

## Data availability statement

The original contributions presented in the study are included in the article/supplementary material, further inquiries can be directed to the corresponding author/s.

## Ethics statement

This study was approved by the Ethics Committee of Liangxiang Hospital. Written informed consent was obtained from all participants for their participation in this study.

## Author contributions

HY and ChaZ designed the study and drafted the manuscript. NW, ChuZ, and SL collected the MRI data. SL and ChuZ analyzed and interpreted the results of the data. ZD and GC revised the manuscript. All authors approved the final manuscript.
